# Setting up a comprehensive management model for chronic conditions/Puesta en marcha de un modelo de gestión integral de procesos crónicos

**Published:** 2012-05-29

**Authors:** Carmen Vercher González, Isabel Hermoso Villalba, Ángeles Cervera Navarro, Mª Asunción López Martínez, Mª José Lloria Cremades

**Affiliations:** Campanar Health Centre, Valencia, Spain; Hospital at Home Service, La Fe Hospital, Valencia, Spain; Hospital at Home Service, La Fe Hospital, Valencia, Spain; Campanar Health Centre, Valencia, Spain; Primary Care Management Team, La Fe Department of Health, Valencia, Spain

**Keywords:** case management, disease management, chronic disease, telenursing, gestión de casos, gestion de enfermedades, enfermedad crónica, tele-enfermería

## Introduction

Nowadays, the management of chronic conditions is one of the greatest challenges facing health systems. The current model of care involves one-off contact with chronic patients when their condition worsens, and they remain invisible to the system until subsequent exacerbations.

The ‘Valencia-La Fe’ Department of Health has launched a comprehensive management model for chronic diseases in order to introduce innovations in the care for these patients, identifying and stratifying their catchment population (Kaiser Permanente model), at the same time as ensuring continuity of care through appropriate coordination and integration of services across the different levels of care.

The aim of the project is to provide comprehensive, proactive, appropriate and safe care to patients with complex chronic conditions who require continuous monitoring. The achievement of this objective relies on strengthening the continuity of care through the structured coordination of care and social resources, promoting health education for patients and their caregivers, and adopting new technologies to ensure that their needs are met. It allows health outcomes and quality of life to be improved through ensuring early detection of exacerbations; providing emotional support for and communication with patients and caregivers; and enhancing prevention measures, health education and self-care. Further, at the end of life, a dignified death should be supported by coordinating and using efficiently the broad range of resources required for the provision of care in the final stages [1–5].

The Primary Healthcare Team is the key player for the management of most chronic patients. A new role has been created in the Department of Health: the nurse case manager (NCM) who coordinates care and undertakes the monitoring of complex chronic patients at a distance. The relationship between primary care doctors and specialists has been strengthened. Protocols, care pathways and guidelines for action have been developed by consensus between experts based on the scientific evidence available [6–9].

## Description of the project:

- ** Inclusion criteria:** based on a list obtained from hospital databases of adults in the catchment area of the Valencia-La Fe Department of Health, the population was stratified and level 3 patients identified (that is, patients with chronic diseases and two or more emergency admissions to the hospital in the previous year) [10].
- ** Patient and caregiver information**: general practitioners (GPs) contact patients and/or their caregivers to tell them about the care programme inviting them to participate and requesting their consent. For those who agree, details of their case (patient personal data and contact telephone numbers) are submitted to the Hospital at Home (HAH) Service and to the nurse case manager. In the case of patients who are hospitalised, the HAH doctor is responsible for their recruitment [11].
- ** Education and Secondary Prevention Programme (ESPP):** the HAH team undertakes a comprehensive assessment of patients, designs personalised care plans, encourages self-care and ensures continuity of care. The HAH schedule for visits is as follows: one visit from a doctor, one visit from a social worker and three visits from nurses.
- For each patient, the HAH doctor prepares an up-to-date medical report, incorporating the nursing reports, and requests a social report, if required. The discharge report should also include the diagnosis code (ICD-9). A copy of this medical report is given to the patient and/or caregiver [12, 13].
- ** Scheduled Remote Monitoring:** the NCM confirms the inclusion of patients on the follow-up programme, updates the nursing data on discharge from the ESPP and checks the HAH doctor’s records. The telephone calls with patients and/or caregivers are scheduled, adjusting the frequency of contact in response to changes in patient condition.
- The NCM also assesses whether objectives are met, reviews and updates the care plan, and adjusts treatment. Records should be kept of all telephone calls. If unforeseen changes are required, the patient’s GP is contacted. Depending on the level of social risk perceived by the social worker, it may also be necessary to coordinate healthcare and social services. Lastly, any healthcare equipment under long-term use at home (such as that for oxygen or aerosol therapy) and orthotic and prosthetic devices should be regularly checked [14–16].
- ** Follow-up of the patient in primary care by nurses and doctors:** the frequency of visits should be adjusted in response to changes in patient condition. If clinical worsening arises and a patient meets the criteria for referral, the primary healthcare team should get in touch with the NCM to arrange transfer to another care provider (HAH, acute care hospital, etc.). The care plan is modified in collaboration with the case manager and data are entered onto the corresponding databases [14, 17].
- ** Self-care:** patients monitor their own vital signs, modify their behaviour and manage their medication [18–20].
- **Scheduled appointments with specialists:** these are provided by HAH doctors for cases with a high level of dependence in activities of daily living (Barthel Index <60) who are unable to attend appointments in a doctor’s consultation room.
- ** Care on demand:** the NCM handles any problems that arise over the telephone or arranges for the necessary referral.

## Results

From 2010 until May of 2011, 166 patients were included on the follow-up programme and of these 85 died during the study period (55 of them at home and 30 in hospital).

The amount of resources used has been reduced: the number of admissions fell from 258 (during the year before to inclusion in the programme) to 74 (during the case management period); and, similarly, the length of stay, hospital bed days falling from 2346 (prior to inclusion) to 470 (under case management).

After the assessment of the initial data, we propose to adopt the following indicators for the monitoring programme:

- Patient and/or caregiver level of satisfaction
- Adherence to the specific integrated care pathway for each condition
- Total number of medical and nursing home visits (scheduled and emergency)
- Number of emergencies handled through the Emergency Call Centre
- Number of attendances in the Emergency Department.

## Conclusions

A multidisciplinary approach should be used for the care of complex chronic patients.

There is a need for proactive identification of patients using appropriate tools.

It is important to identify non-cancer patients who require palliative care by using tools that assess the risk of death.

Although further research is required, our type of case management programme seems able to reduce the use of hospital resources (in terms of admissions and inpatient bed days), as well as being a cost-effective way to improve patient quality of life.

## Conference abstract Spanish

## Introducción

El manejo de las condiciones crónicas es uno de los grandes retos a los que se enfrentan los sistemas de salud. El modelo de atención actual mantiene un contacto puntual con los pacientes crónicos cuando su estado empeora, desapareciendo del sistema hasta una nueva descompensación.

El Departamento de Salud Valencia La Fe, ha puesto en marcha un Modelo de Gestión integral de Enfermedades Crónicas para innovar la atención de estos pacientes, identificando y estratificando su población de referencia (modelo Kaiser Permanente) al mismo tiempo que garantiza la continuidad de sus cuidados mediante la adecuada coordinación e integración de los diferentes niveles asistenciales.

La intervención tiene como objetivo proporcionar atención integral, proactiva, apropiada y segura a los pacientes con condiciones crónicas complejas que precisan de un control continuado en el tiempo, potenciando la continuidad asistencial a través de una coordinación estructurada de la atención sanitaria existente y los recursos sociales, impulsando la educación sanitaria del paciente y cuidador, e incorporando nuevas tecnologías para asegurar la cobertura de las necesidades del paciente y cuidador, mejorando los resultados en salud y calidad de vida, detectando precozmente las descompensaciones, proporcionando apoyo emocional y comunicación al paciente y cuidador, potenciando las medidas de prevención, educación sanitaria y el autocuidado, garantizando una muerte digna, si ocurre y coordinando los diversos recursos implicados en la atención con una utilización eficiente de los mismos [1–5].

El equipo de Atención Primaria será el actor clave para la gestión de la mayoría de los pacientes crónicos. Se incorpora una nueva figura, la **enfermera gestora de casos (EGC) del** **Departamento** que coordina los cuidados y lleva a cabo el seguimiento a distancia de los pacientes crónicos complejos. Se potencia la relación mantenida entre los médicos de atención primaria y los médicos de las diferentes especialidades. Se elaboran por consenso con especializada, **protocolos, rutas asistenciales y guías** de actuación basadas en la evidencia científica [6–9].

## Descripción de la experiencia:

- ** Criterios de inclusión:** Se estratificó a la población, identificando a los pacientes de Nivel 3 a partir del listado obtenido de las bases de datos hospitalarias de aquellos pacientes adultos pertenecientes al Departamento La Fe, con enfermedades crónicas y con 2 ó mas ingresos urgentes en el último año [10].
- ** Informar al paciente y cuidador:** El médico de AP contacta con el paciente y cuidador y le informa del programa, confirma la voluntariedad del paciente para su inclusión; remite la propuesta a la UHD (Unidad de Hospitalización Domiciliaria) y contacta telefónicamente con la gestora de casos para realizar la propuesta de inclusión en gestión de casos proporcionando los datos del paciente y un teléfono de contacto. Si el paciente está hospitalizado, será el médico de UHD el que incluirá al paciente [11].
- ** Programa de Educación y Prevención Secundaria (PEPS**): El equipo de la UHD realiza la valoración integral del paciente, elabora el plan de cuidados, impulsa el autocuidado y asegura el *continuum* asistencial. El plan de visitas de la UHD se establece del siguiente modo: 1 visita médica, 1 visita de trabajadora social y 3 visitas de enfermería.
- El médico de la UHD elabora el informe médico actualizado unificando su valoración y la de enfermería e indicando si precisa informe social. Registra el alta del paciente y codifica los diagnósticos (CIE-9). Este informe clínico se entrega al paciente y cuidador [12, 13].
- ** Seguimiento a distancia programado:** la EGC comprueba la inclusión del paciente en Seguimiento Programado, actualiza la información de enfermería al alta del PEPS, comprueba el registro del médico de UHD del alta del PEPS, programa el contacto telefónico con el paciente y/o cuidador, adecua la frecuencia en función de los cambios que sufra el paciente.
- La EGC también valora el cumplimiento de objetivos, revisa y actualiza el plan de cuidados, ajusta el tratamiento, registra el contacto telefónico. Si el paciente precisa modificaciones no previstas consulta telefónicamente con el médico de AP. Coordina con recursos socio-sanitarios, en función de criterios de riesgo social detectados por la trabajadora social. Controla el equipamiento sanitario domiciliario de larga duración (oxigenoterapia, aerosolterapia, etc.) y material ortoprotésico [14–16].
- ** Seguimiento por Atención Primaria:** enfermería y médico. La frecuencia de visitas puede modificarse en función de los cambios que sufra el paciente. Contacta telefónicamente con la EGC si el paciente cumple criterios de derivación por descompensación a otro recurso asistencial implicado en la atención (UHD, Hospital de agudos, etc.). Ajusta el plan de atención con la Gestora de Casos. Cumplimenta registros [14, 17].
- ** Autocuidado:** el paciente monitoriza signos vitales, modificación de la conducta y manejo de medicación [18–20].
- **Consulta Programada de Atención Especializada:** Esta tarea la realizará el médico de UHD en los casos de pacientes con alta dependencia para las actividades básicas de la vida diaria (I. Barthel <60) que no puedan desplazarse a la consulta.
- ** Atención a la demanda**: la EGC resuelve telefónicamente la consulta o procede a su derivación.

## Resultados

Se han incluido en el programa 166 pacientes (año 2010) que se han seguido hasta mayo del 2011. Ha habido 85 exitus, 55 en su domicilio, y 30 en el hospital.

El consumo de recursos global ha disminuido, de 258 ingresos (365 días previos a la inclusión) a 74 ingresos (durante la gestión de casos), así mismo han disminuido las estancias (2346 días previos a la inclusión frente a 470 durante la gestión de casos).

Tras la valoración de los datos obtenidos, nos planteamos incluir en el programa la monitorización de:

- El grado de satisfacción del paciente y del cuidador.
- La adherencia al Proceso Clínico Integrado específico para la enfermedad
- Número total de visitas domiciliarias médicas y de enfermería (programadas y urgentes)
- Número de urgencias atendidas por el CICU (Centro de Información y Coordinación de Urgencias)
- Número de atenciones en Urgencia.

## Conclusiones

El abordaje de los pacientes crónicos complejos, debe ser multidisciplinar.

Es necesario mejorar la identificación de pacientes, con la utilización de herramientas que permitan su identificación proactiva.

Es importante la identificación de paciente paliativo no oncológico mediante la utilización de herramienta de detección de riesgo de mortalidad.

Pendiente de más estudios, la gestión de casos permite reducir el consumo de recursos hospitalarios (ingresos, estancias…) y la calidad de vida de los pacientes con un coste eficiente.
